# Supplementary material for: Relationship between Corticospinal Excitability While Gazing at the Mirror and Motor Imagery Ability
Source: Brain Sci. 2023 Mar 9;13(3):463. doi: 10.3390/brainsci13030463 (PMC10046091; doi:10.3390/brainsci13030463)
Supplement: Supplementary file 1 [file brainsci-13-00463-s001.zip › brainsci-2238703-supplementary.pdf]

Supplementary Material

Supplementary table S1. Evaluation results of motor imagery ability

Participants	age	Sex	KVIQ		Mental chronometry (MC)		
			Visual imagery	Kinesthetic imagery	Imaged time (s)	Actual time (s)	MC score
A	21	Female	50	49	6.04	10.98	4.94
B	22	Male	39	40	8.19	11	2.81
C	21	Male	41	34	16.4	12.52	3.88
D	21	Female	45	46	10.56	9.9	0.66
E	24	Male	39	38	5.75	10.28	4.53
F	23	Female	40	28	8.44	13.11	4.67
G	24	Female	44	46	7.98	8.41	0.43
H	21	Female	41	36	16.51	14.82	1.69
I	21	Female	37	39	5.28	9.09	3.81
J	22	Female	46	46	8.73	10.32	1.59
K	22	Female	47	36	8.76	9.91	1.15
L	22	Female	48	47	10.22	9	1.22
M	22	Male	49	47	7.45	9.89	2.44
N	22	Female	43	33	4.47	8.85	4.38
O	21	Female	31	31	8.99	9.41	0.42
P	20	Female	28	35	8.09	9.49	1.4
Q	22	Female	40	40	6.9	9.95	3.05
R	22	Female	40	38	5.61	9.47	3.86
S	21	Male	48	38	8.46	9.24	0.78
T	21	Female	50	47	17.98	16.24	1.74
U	22	Male	48	35	9.44	12.51	3.07
V	23	Female	49	39	6.61	12.37	5.76
W	21	Male	49	45	9.15	12.4	3.25
X	20	Female	48	37	7.1	9.57	2.47

KVIQ, kinesthetic and visual imagery questionnaire; MC score, absolute difference between imaged time and actual time

Supplementary table S2. MEP measurements at rest and while gazing at the mirror

Participants	age	Sex	MEP rest (mV)	MEP gazing at the mirror (mV)	MEP ratio
A	21	Female	0.29	1.03	3.58
B	22	Male	0.38	0.81	2.14
C	21	Male	0.48	0.76	1.58
D	21	Female	0.41	1.3	3.18
E	24	Male	0.16	0.33	2.15
F	23	Female	0.08	0.07	0.83
G	24	Female	0.1	0.49	4.92
H	21	Female	0.15	0.34	2.21
I	21	Female	0.16	0.25	1.59
J	22	Female	0.05	0.29	5.38
K	22	Female	0.19	0.67	3.61
L	22	Female	0.1	0.36	3.53
M	22	Male	0.24	0.63	2.59
N	22	Female	0.25	0.1	0.42
O	21	Female	0.19	0.55	2.88
P	20	Female	0.31	0.75	2.46
Q	22	Female	0.12	0.28	2.3
R	22	Female	0.1	0.61	6
S	21	Male	0.25	1.5	6.1
T	21	Female	0.02	0.1	6
U	22	Male	0.12	0.34	2.9
V	23	Female	0.21	0.67	3.2
W	21	Male	0.1	0.57	6
X	20	Female	0.28	0.32	1.1

MEP, motor evoked potential; MEP ratio, calculated by dividing MEP while gazing at the mirror by MEP at rest
